# Islet Cell Antibodies Represent Autoimmune Response Against Several Antigens

**DOI:** 10.1155/EDR.2001.85

**Published:** 2001

**Authors:** Lisa Månsson, Carina Törn, Mona Landin-Olsson

**Affiliations:** 1 lnstitution of Medicine Lund University Hospital Lund S-221 85 Sweden; 2 Department of Medicine Lund University Hospital Lund S-221 85 Sweden

## Abstract

To study the antigens involved in the islet cell antibody (ICA) reaction we selected 30 patient serum samples (ten in each group) positive for ICA and one other additional autoantibody, such as glutamic acid decarboxylase antibodies (GADA), thyrosine phosphatase antibodies (IA-2A) or insulin autoantibodies (IAA). The serum samples were incubated with the specific antigen (GAD65, IA-2 or insulin) and the ICA analysis and the corresponding immunoprecipitation assay were performed before and after the absorption.

We could then demonstrate that specific autoantibodies against GAD65 and IA-2 could be absorbed with the corresponding antigen, since ten GADA positive and six IA-2A samples turned completely negative. However, the ICA reaction after absorption with GADA, IA-2A and insulin was still present, although at significantly lower levels. The results strongly indicate that the ICA reaction represents simultaneous autoimmunity against several other antigens beside GAD65, IA-2 and insulin.


*Int. Jnl. Experimental Diab. Res.*, Vol. 2, pp. 85–90
Reprints available directly from the publisher
Photocopying permitted by license only

© 2001 OPA (Overseas Publishers Association) N.V.
Published by license under
the Harwood Academic Publishers imprint,
part of Gordon and Breach Publishing,
a member of the Taylor & Francis Group.
Printed in the U.S.A.

### Islet Cell Antibodies Represent Autoimmune Response Against Several Antigens

LISA MÅNSSON[a], CARINA TÖRN[a] and MONA LANDIN-OLSSON[b,*]

[a]*Institution of Medicine,* [b]*Department of Medicine, Lund University Hospital, S-221 85 Lund, Sweden*

*(Received 24 March 2000; In final form 2 January 2001)*

**To study the antigens involved in the islet cell antibody (ICA) reaction we selected 30 patient serum samples (ten in each group) positive for ICA and one other additional autoantibody, such as glutamic acid decarboxylase antibodies (GADA), thyrosine phosphatase antibodies (IA-2A) or insulin autoantibodies (IAA). The serum samples were incubated with the specific antigen (GAD65, IA-2 or insulin) and the ICA analysis and the corresponding immunoprecipitation assay were performed before and after the absorption.**

**We could then demonstrate that specific autoantibodies against GAD65 and IA-2 could be absorbed with the corresponding antigen, since ten GADA positive and six IA-2A samples turned completely negative. However, the ICA reaction after absorption with GADA, IA-2A and insulin was still present, although at significantly lower levels. The results strongly indicate that the ICA reaction represents simultaneous autoimmunity against several other antigens beside GAD65, IA-2 and insulin.**

*Keywords*: Diabetes mellitus; Autoantibodies; Islet cell antibodies; Glutamic acid decarboxylase antibodies; Thyrosine phosphatase antibodies; Insulin autoantibodies

### INTRODUCTION

Type 1 diabetes has been regarded as an autoimmune disease since islet cell antibodies (ICA) were described in diabetic patients in 1974.[1] The immunofluorescense method for ICA do not define the antigen and the search for the antigen responsible for this reaction started. Insulin was one strong candidate and autoantibodies against insulin were later also demonstrated in Type 1 diabetic patients.[2] Insulin autoantibodies (IAA) were soon followed by the discovery of several other autoantibodies.[3] Glutamic acid decarboxylase antibodies (GADA)[4] and thyrosine phosphatase antibodies (IA-2A)[5,6] are two of the most frequently occuring autoantibodies together with IAA. Beside pure proteins like GAD65, IA-2 and others, a sialic acid containing glycolipid[7] has been shown to be another antigen in autoimmune diabetes. However, it has been suggested that most of ICA's reactivity is represented by GADA[8] and IA-2A.[5,9]

From a large population based study,[10] we had the possibility to select groups of samples with a combination of ICA positivity together with an isolated positivity for either GADA, IA-2A or IAA. The aim was to study to which extent the ICA reaction could be blocked by an absorption to the specific antigen (GAD65, IA-2

*Corresponding author. Tel.: +46 +46-171000, Fax: +46+46-2110908, e-mail: mona.landin-olsson@med.lu.se or insulin). This would clarify if GAD65, IA-2 and insulin respectively were the main antigens that contribute to the ICA reaction or if other antigens have to be taken into account.

### MATERIALS AND METHODS

### Materials

Samples were selected from The Diabetes Incidence Study in Sweden (DISS), a nationwide study including 764 newly diagnosed diabetic patients in the ages 15–34 years.[10] We selected 30 samples, all positive for ICA and in addition positive for one more autoantibody (GADA, IA-2A or IAA). In the first group in which positivity for ICA and GADA was required, 61 samples fulfilled the criteria and ten were randomly selected. In the ICA and IA-2A positive group, 26 samples were available and ten were randomly selected. In the third group, only three samples were exclusively positive for only ICA and IAA, and a weak GADA positivity was therefore allowed in the remaining seven samples. The study was approved by the Ethical Committees at all University Hospitals in Sweden (Stockholm, Göteborg, Linköping, Lund, Umeå and Uppsala).

### Assays

ICA were analysed by indirect immunofluorescence on unfixed human pan-creas from an organ donor with bloodgroup 0 [11]. Levels were expressed as Juvenile Diabetes Foundation Units (JDF-U) and the lower limit for positivity was 4 JDF-U. All analyses were performed in a blinded format.

GADA were analysed in a radioimmunoprecipitation assay using in vitro translated human GAD65 labelled with $^{35}$S-methionine.[12] Levels were expressed as an index calculated in relation to a positive and negative standard sample. An index below 0.08 was considered as negative.

IA-2A were analysed in a radioimmunoprecipitation assay similar to the GADA-assay.[13] An index below 0.05 was considered as negative.

IAA were analysed by radioimmunoassay and the samples were also displaced by cold insulin to avoid unspecific binding.[2] A specific Δ-binding percent below 0.8 was considered as negative.

### Preparation of Antigens

GAD65 and IA-2 were prepared using the TNT® Coupled Reticulocyte Lysate System (Promega, Madison, USA) for *in vitro* transcription-translation.[12,13] An approximation of the obtained protein concentration was done by using the Luciferase Assay System (Promega, Madison, USA). A standard curve of light units versus enzyme concentration was produced with a luminometer giving an indirect measurement of the protein concentration. From previous performed ELISA assays of GADA, we could approximate that an amount of 40–180 ng cold antigen for each millilitre of test serum would be needed to bind the autoantibodies. Since insulin in the form of the commercial insulin Actrapid® (Novo Nordisk, Denmark), was contrary to GAD65 and IA-2, available in unlimited amounts it was added in excess (3.5mg/ml) to the IAA positive samples. One samples was reanalysed after absorption with half insulin and half serum.

### Absorption

Each serum sample was divided in two aliquotes, one was incubated with the cold antigen, as described above, for 12 hours at 4°C, and the other was used as a control. Both aliquotes were equally diluted and reanalysed for ICA and for the specific antibody (GADA or IA-2A) by the immunoprecipitation assay. Since cold insulin was available in unlimited amounts and therefore added in excess, the IAA positive samples were only reanalysed for

ICA but not for IAA. To test if immunoreactive insulin was available in the tissue samples, a pancreas section was incubated with an anti-insulin antibody from mouse (mouse anti-human insulin IgG, Santa Cruz Biotechnology, USA). In this experiment immunoreactive insulin was detected by a Texas red labelled anti-mouse IgG.

Due to dilutions during the absorption procedure, ICA levels below 29 JDF units could not be determined after absorption. To test the absorption procedure as such, some samples were retested after incubation with bovine serum albumin (BSA) as an irrelevant antigen.

### Statistical Analyses

The Spearman rank correlation test ($r_s$) was used to test for correlations between ICA and the other antibodies. Levels of autoantibodies before and after absorption were compared with Wilcoxon signed rank test. A p-value less than 0.05 was considered as significant. Stat View 4.5 for Macintosh was used.

### RESULTS

Among these highly selected samples, no significant correlation could be found between ICA and the other antibodies, GADA, IA-2A or IAA. In the group of ICA and GADA positive samples, GADA could be absorbed since the levels were significantly lower ($p < 0.005$), and all samples were considered GADA negative after absorption with GAD65. Of these samples, eight of ten showed a reduction in ICA level ($p = 0.01$), corresponding to one or two dilution steps (Fig. 1). However, eight of the ten samples were still ICA positive, and the remaining two had a level below 29 JDF units but exact ICA level could not be determined, due to prior dilution during the absorption procedure. Anyhow, both these were at least two dilution steps lower than before absorption. In the group positive for ICA and IA-2A, four samples were still weakly IA-2A positive after absorption with IA-2, while IA-2A were abolished in the others. The decreases in levels were significant ($p = 0.005$). Nine of ten had decreased their ICA levels with at least one

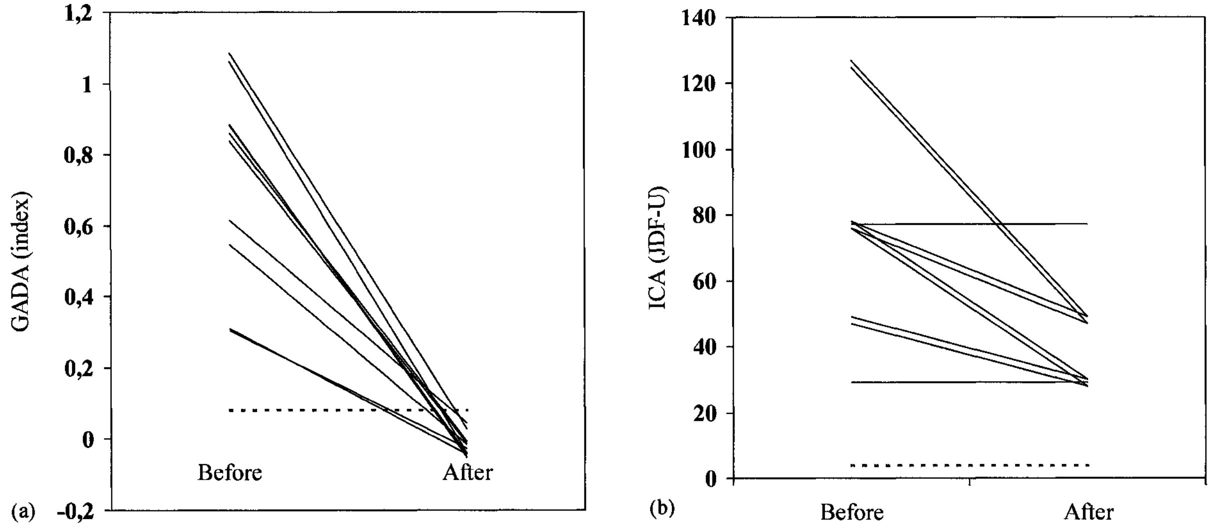

FIGURE 1 In the group with ten samples positive for ICA and GADA the initially positive GADA levels totally disappeared after absorption with cold GAD65 (a). Index below 0.08 was considered as negative and indicated by a dashed line. Corresponding analyses for ICA showed a significant but not complete decrease in ICA levels after absorption with GAD65 (b). For two of the samples, the lowest JDF-U was not possible to determine due to dilution during the absorption procedure. The cut off level of 4 JDF-U is indicated with a dashed line.

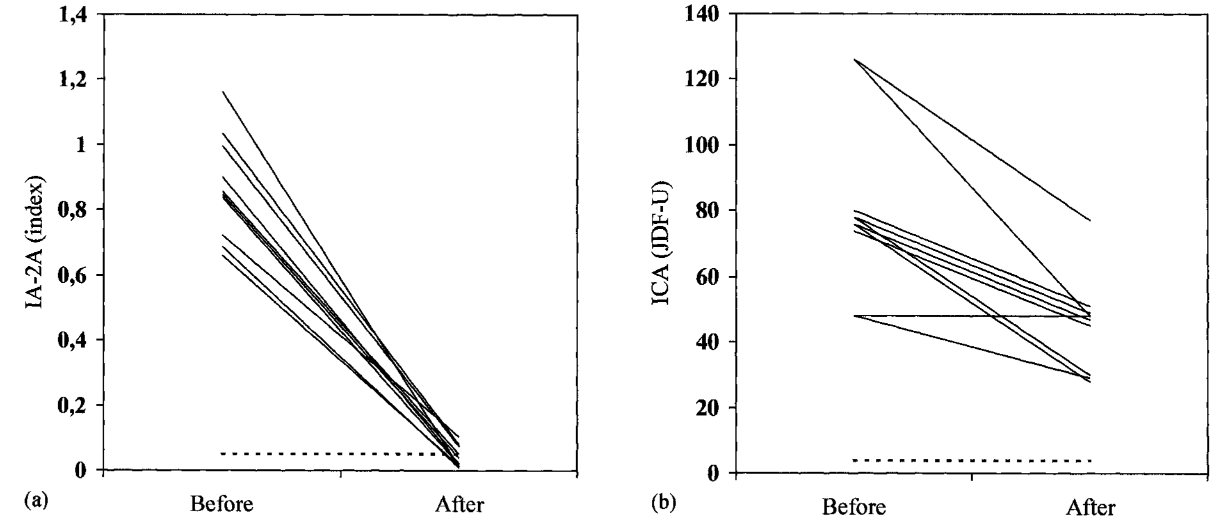

FIGURE 2 In the group positive for ICA and IA-2A, a remaining weak IA-2A positivity could be demonstrated in four of the ten samples despite absorption with IA-2 (a). Index below 0.05 was considered as negative and is indicated by a dashed line. Decreased ICA levels were found after absorption with IA-2 (b). For two of the samples, the lowest JDF-U was not possible to determine due to dilution during the absorption procedure. The cut off level 4 JDF-U is shown by a dashed line.

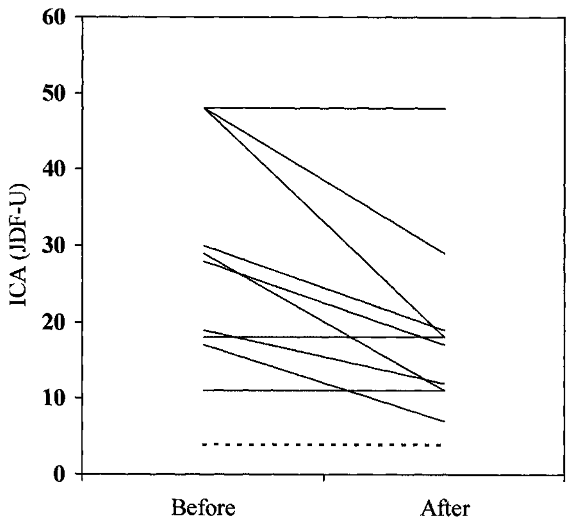

FIGURE 3 In the ICA and IAA positive group, seven of ten samples showed a decrease in ICA level. The cut off level 4 JDF-U is shown by the dashed line.

or two ICA titer steps ($p = 0.01$) (Fig. 2), although the lowest ICA levels not could be determined in two samples due to prior dilutions. None of the eight end point titrated samples turned completely negative for ICA. In the ICA and IAA positive group, seven of ten showed a decrease in ICA level ($p = 0.005$) after insulin absorption, but none became totally ICA negative (Fig. 3). One of the samples positive for ICA and IAA was reanalysed after insulin added in dilution half insulin and half serum, despite this the ICA reaction was still positive. The pancreas section incubated with an anti-insulin antibody turned out with a positive immunofluorescence, confirming that immunoreactive insulin was available in the tissue sections. The sample incubated with BSA, an irrelevant antigen, did not change the levels of GADA or ICA.

### DISCUSSION

The study showed that ICA were not easily abolished by a single absorption with one specific antigen, even if the antigen specific antibody disappeared. This finding indicate that several autoantibodies others than GADA, IA-2A and IAA are simultaneously present in Type 1 diabetes, even if there is a dominance of one of these autoantibodies. Many studies suggest that GAD65, IA-2 and insulin are the major antigens responsible for the ICA reaction.[8,9] Since ICA is such a time consuming and in many aspects complicated assay, a combination of the immunoprecipitation analyses for GADA and IA-2A have been suggested to replace the ICA method.[14,15] Our results indicate that several other antigens not tested in this study, are involved in the ICA reaction.

Correlations between ICA levels and levels of GADA and IA-2A have been demonstrated in the entire cohort from which we did the original selection.[10] However, in this subset of samples no correlation could be found between ICA and the other autoantibodies (GADA, IA-2A or insulin). This could be due to the small number of samples and to the fact that they were highly selected.

In this study we could show an almost complete block of GADA and IA-2A after absorption with the corresponding specific antigen, GAD65 and IA-2. However, in four of ten IA-2A positive samples, IA-2A could still be detected after absorption but in very low levels. This could be due to an incubation with insufficient amount of antigen, since all four samples had relatively high initial level of IA-2A, the amount of antigen was limited and the needed amount for absorption could only be approximised. However, against this could be argued that the sample positive for ICA and IAA that was reanalysed with insulin in excess was still ICA positive.

The specific autoantibodies are not evenly represented among the diabetic population, since for example GADA is more frequent in females and in higher ages, while IAA are more frequent in young children.[16] ICA seems to be more evenly distributed among type 1 diabetic patients, despite a higher frequency among children compared with adults,[17] probably reflecting the general autoimmunity among these patients rather than gender or age specificity for the antibody. Our hypothesis is that different exogenous triggers, depending on different environmental factors related to age, gender and life exposure, could start an immune response. This could, in susceptible subjects, lead to a general activation and hyperreactivity against an increasing number of beta cell specific autoantigens. The autoimmune response could also spread outside the beta cells since in fact other autoimmune diseases are more frequent among diabetic patients.[18] In others, the initial immune response may be self limiting and never proceed to clinical signs of diabetes. This is supported by the observed low predictability of diabetes in healthy subjects positive for only one autoantibody and the increasing risk for diabetes with multiple autoantibody positivity.[19]

In conclusion, both GADA, IA-2A and IAA are partly responsible for the ICA reaction in sera from patients positive for ICA. However, if ICA's reactivity was constituted only of these autoantibodies the ICA reaction would be blocked more completely than what was found in our study. Our results indicate a more extended heterogeneity of the contribution to ICA positivity and probably simultaneous involvement of several other beta cell specific autoantigens beside GAD65, IA-2 and insulin.

### *Acknowledgements*

This study was possible by a scholarship (Lisa Månsson) from the Medical Faculty, Lund University. The study also received grants from Stig Alméns Foundation.

The members of the Diabetes Incidence Study in Sweden (DISS) group are thanked; Hans Arnqvist, Elisabeth Björk, Göran Blohmé, Jan Bolinder, Jan Eriksson, Bengt Littorin, Lennarth Nyström, Göran Sundkvist and Jan Östman. Dr George Eisenbarth, Denver, Colorado is thanked for kindly donating the plasmid pICA512 bdc and Dr Åke Lernmark, Seattle, Washington for donating the plasmid pGADcDNAII. Mrs Birgitta Persson is acknowledged for expert technical assistance.

### *References*

[1] Bottazzo, C. F., Florin-Christensen, A. and Doniach, D. (1974). Islet-cell antibodies in diabetes mellitus with autoimmune polyendocrine deficiencies, *Lancet*, **2**, 1279–1283.

[2] Palmer, J. P., Asplin, C. M., Clemons, P., Lyen, K., Tatpati, O., Raghu, P. K. and Paquette, T. L. (1983). Insulin antibodies in insulin-dependent diabetics before insulin treatment, *Science*, **222**, 1337–1339.

[3] Atkinson, M. A. and Maclaren, N. K. (1993). Islet cell autoantigens in insulin-dependent diabetes, *J. Clin Invest*, **92**, 1608–1616.

[4] Baekkeskov, S., Aanstoot, H. J., Christgau, S., Reetz, A., Solimena, M., Cascalho, M., Folli, F., Richter-Olesen, H. and Camilli, P. (1990). Identification of the 64K autoantigen in insulin-dependent diabetes as the GABA-synthesizing enzyme glutamic acid decarboxylase, *Nature*, **347**, 151–156.

[5] Bonifacio, E., Lampasona, V., Genovese, S., Ferrari, M. and Bosi, E. (1995). Identification of protein tyrosine phosphatase-like IA-2 (Islet cell antigen 512) as the insulin-dependent diabetes-related 37/40K autoantigen and a target of islet-cell antibodies, *J. Immunol*, **155**, 5419–5426.

[6] Rabin, D. U., Pleasic, S. M., Shapiro, J. A., Yoo-Warren, H., Oles, J., Hicks, J. M., Goldstein, D. E. and Rae, P. M. M. (1994). Islet cell antigen 512 is a diabetes-specific islet autoantigen related to protein tyrosine phosphatases, *J. Immunol*, **152**, 3183–3188.

[7] Nayak, R. C., Omar, M. A. K., Rabizadeh, A., Srikanta, S. and Eisenbarth, G. S. (1985). "Cytoplasmic" islet cell antibodies. Evidence that the target antigen is a sialoglycoconjugate, *Diabetes*, **34**, 617–619.

[8] Atkinson, M., Kaufman, D., Newman, D., Tobin, A. and Maclaren, N. (1993). Islet cell cytoplasmic autoantibody reactivity to glutamate decarboxylase in insulin-dependent diabetes, *J. Clin Invest*, **91**, 350–356.

[9] Myers, M., Rabin, D. U. and Rowley, M. J. (1995). Pancreatic islet cell cytoplasmic antibody in diabetes is represented by antibodies to islet cell antigen 512 and glutamic acid decarboxylase, *Diabetes*, **44**, 1290–1295.

[10] Landin-Olsson, M., Arnqvist, H., Blohmé, G., Lernmark, Å., Lithner, F., Littorin, B., Nyström, L., Scherstén, B., Sundqvist, G., Wibell, L. and Östman, J. (1997). Clinical versus laboratory classification of diabetes type in young adults, *Diabetologia*, **40**, suppl 1:I–V, A76.

[11] Landin-Olsson, M., Sundkvist, G. and Å. Lernmark, (1987). Prolonged incubation in the two-colour immunofluorescence test increases the prevalence and titres of islet cell antibodies in type 1 (insulin-dependent) diabetes mellitus, *Diabetologia*, **30**, 327–332.

[12] Grubin, C. E., Daniels, T., Toivola, B., Landin-Olsson, M., Hagopian, W. A., Li, L., Karlsen, A. E., Boel, E., Michelsen, B. and Lernmark, Å. (1994). A novel radioligand binding assay to determine diagnostic accuracy of isoform-specific glutamic acid decarboxylase antibodies in childhood IDDM, *Diabetologia*, **37**, 344–350.

[13] Gianani, R., Rabin, D. U., Verge, C. F., Yu, L., Babu, S. R., Pietropaolo, M. and Eisenbarth, G. S. (1995). ICA512 autoantibody radioassay, *Diabetes*, **44**, 1340–1344.

[14] Wiest-Ladenburger, U., Hartmann, R., Hartmann, U., Berling, K., Böhm, B. O. and Richter, W. (1997). Combined analysis and single-step detection of GAD65 and IA2 autoantibodies in IDDM can replace the histochemical islet cell antibody test, *Diabetes*, **46**, 565–571.

[15] Månsson, L., Törn, C., Landin-Olsson, M. and Lernmark, Å. The Diabetes Incidence Study Group in Sweden (1998). Can the laborious ICA assay be replaced by analyses of other pancreatic antibodies?, *European J. of Endocrinology*, **138**, supplement 1,15:44.

[16] Vandewalle, C. L., Falorni, A., Svanholm, A., Lernmark, Å., Pipeleers, D. G. and Gorus, F. K. The Belgian Diabetes Registry. (1995). High diagnostic sensitivity of glutamate decarboxylase autoantibodies in insulin-dependent diabetes mellitus with clinical onset between age 20 and 40 years, *J. Clin Endocrinol Metab*, **80**, 846–851.

[17] Hagopian, W., Sanjeevi, C., Kockum, I., Landin-Olsson, M., Karlsen, A., Sundkvist, G., Dahlquist, G., Palmer, J. and Lernmark, Å. (1995). Glutamte decarboxylase-, insulin-, and islet cell antibodies and HLA typing detect diabetes in a general population-based study of swedish children, *J. Clin Invest*, **95**, 1505–1511.

[18] Drell, D. W. and Notkins, A. L. (1987). Multiple immunological abnormalities in patients with type 1 (insulin-dependent) diabetes mellitus, *Diabetologia*, **30**, 132–143.

[19] Verge, C. F., Gianani, R., Kawasaki, E., Yu, L., Pietripaolo, M., Jackson, R. A., Chase, H. P. and Eisenbarth, G. S. (1996). Prediction of type I diabetes in first-degree relatives using a combination of insulin, GAD, and ICA512bdc/IA-2 autoantibodies, *Diabetes*, **45**, 926–933.

